# Short-lead seasonal precipitation forecast in northeastern Brazil using an ensemble of artificial neural networks

**DOI:** 10.1038/s41598-023-47841-y

**Published:** 2023-11-22

**Authors:** Enzo Pinheiro, Taha B. M. J. Ouarda

**Affiliations:** Centre Eau-Terre-Environnement, Institut National de la Recherche Scientifique, 490 de la Couronne, Office 2435, Québec, QC G1K9A9 Canada

**Keywords:** Atmospheric dynamics, Ocean sciences

## Abstract

This study assesses the deterministic and probabilistic forecasting skill of a 1-month-lead ensemble of Artificial Neural Networks (EANN) based on low-frequency climate oscillation indices. The predictand is the February-April (FMA) rainfall in the Brazilian state of Ceará, which is a prominent subject in climate forecasting studies due to its high seasonal predictability. Additionally, the study proposes combining the EANN with dynamical models into a hybrid multi-model ensemble (MME). The forecast verification is carried out through a leave-one-out cross-validation based on 40 years of data. The EANN forecasting skill is compared with traditional statistical models and the dynamical models that compose Ceará’s operational seasonal forecasting system. A spatial comparison showed that the EANN was among the models with the smallest Root Mean Squared Error (RMSE) and Ranked Probability Score (RPS) in most regions. Moreover, the analysis of the area-aggregated reliability showed that the EANN is better calibrated than the individual dynamical models and has better resolution than Multinomial Logistic Regression for above-normal (AN) and below-normal (BN) categories. It is also shown that combining the EANN and dynamical models into a hybrid MME reduces the overconfidence of the extreme categories observed in a dynamically-based MME, improving the reliability of the forecasting system.

## Introduction

Seasonal climate forecasting relies on the interactions of the atmosphere with slower components of the climate system, which yields modes of variability that have either a quasi-periodic evolution or a large persistence^1^. The most well-known example is the El Niño–Southern Oscillation (ENSO), an ocean–atmosphere phenomenon in the equatorial Pacific Ocean with a periodicity of approximately four years, responsible for noteworthy atmospheric and oceanic variations in several regions of the globe^2^. ENSO teleconnections have been extensively studied; for instance, the phenomenon is associated with precipitation anomalies over large regions of Canada^3^, in the United Arab Emirates (UAE)^4,5^, in northern Tunisia^6^, in northern and southern Brazil^7^, among other regions. Other large-scale climate oscillations, such as the North Atlantic Oscillation^8^ or the Indian Ocean Dipole^9^, are also known to have impacts worldwide.

The physical processes that bridge widely separated regions involve complex interactions of the Earth system components^10^, yielding response times that range from weeks to several months ahead^11,12^. Therefore, climate oscillations are powerful predictors and have been employed for empirical forecasting in several regions. In this context, artificial neural networks (ANN), universal approximation functions used for deriving unknown relationships between the variables of interest, have been widely used for long-term forecasting of hydroclimatic variables. For instance, a recurrent neural network based on climate indices was employed to forecast annual regional runoff, in terms of potential energy inflow, in northern Quebec and the Labrador region^13^. This study demonstrated that using the Baffin Island-West Atlantic, an index that describes the temporal evolution of the Canadian Polar Trough, and Pacific-North American (PNA) indices improved the forecast ability compared to a nil scenario where only energy inflows are used. Moreover, a multi-layer perceptron ANN was employed for modelling the spring rainfall in Victoria, southeastern Australia, using lagged ENSO indices and the Dipole Mode Index^14^. The results showed that the ANN model resulted in lower errors than multiple linear regression for the region. Another study carried out for short-to-long-term monthly rainfall forecasting in southeastern Australia showed that individual optimization of ANN models for each calendar month yields better results than conducting an optimization for all months together^15^. Recently, a nonlinear canonical correlation analysis based on ANN was employed to model the relationship between global climate indices and monthly wind speed in the UAE^16^. They showed that the model predicted the monthly values with a relative error of around 5%. Another recent study evaluated five types of ANNs for monthly rainfall forecasting in Suez, Egypt^17^. They showed that a general regression neural network represented better the rainfall variability and yielded more accurate forecasts than the other ANNs for the studied area.

Ensemble learning is the process of training multiple prediction models to achieve the same task, which are combined to make a final prediction^18^. Due to the improvement in computing capacity, ensemble learning techniques have become popular in hydroclimate forecasting studies. Ensemble models have the advantage of being more stable and provide a better generalization ability than single models^19,20^. Additionally, such models allow quantifying the modelling uncertainties. A Canonical Correlation Analysis (CCA)-based ensemble of ANNs (EANN) model was proposed for flood quantile estimates at ungauged sites^21^. The authors showed that the CCA-EANN outperformed the accuracy of several other statistical methods, including a CCA-single ANN model. To address the rainfall forecasting problem, an EANN, optimized through the Particle Swarm algorithm and using climate variables (sea surface temperature, geopotential height and air temperature) as predictors, was employed to forecast the April mean rainfall from 37 weather stations in Guangxi, China^22^. The deterministic evaluation showed that the proposed model yielded more accurate predictions than multiple linear and stepwise regressions. Nevertheless, the study did not assess the probabilistic performance of the EANN for climate forecasting. A recent study^23^ evaluated the deterministic and probabilistic forecasting skills of an ensemble of machine-learning models based on climate indices for seasonal precipitation forecasting in China. They concluded that the machine learning multi-model ensemble (MME) outperformed the North American Multi-Model Ensemble (NMME) in terms of deterministic and probabilistic performance for several lead times. Although this study provided a probabilistic evaluation of the proposed MME, only model accuracy was evaluated, leaving out other important attributes of probabilistic forecasting, such as reliability and resolution, which are key to any operational forecasting system.

Northeastern Brazil is a prominent subject in seasonal forecasting because its climate variability is strongly driven by large-scale climate oscillations, resulting in high predictability^24,25^. The Atlantic Intertropical Convergence Zone (ITCZ) is the major meteorological system responsible for the northeastern Brazil precipitation regime during austral fall^26^. On an interannual timescale, the ITCZ positioning is mainly controlled by an interhemispheric gradient of sea surface temperature (SST) anomalies in the tropical Atlantic, which in turn is formed by two modes of variability, i.e., the Tropical North (TNA) and South (TSA) Atlantic modes^27–29^. Evidence suggests interdependency between the two modes^30^ modulated by a feedback process between wind, evaporation and SST (WES)^31^, which maintains SST anomalies in the deep tropics during austral fall. Its life cycle is marked by an initial development in summer, peaking during fall and decaying afterwards^32^.

Adjustments in the Walker circulation during ENSO years lead to vertical motion anomalies in a large area in northeastern Brazil, thus directly influencing the convective activity over the region^33^. Furthermore, changes in the tropical Pacific deep convection excite the PNA pattern^34^, which in turn impacts the air subsidence over the North Atlantic Subtropical High^35^. In response, northeasterly trade anomalies are observed in the tropical North Atlantic, forcing the development of the TNA mode^27,35,36^. Evidence shows that an increase in ENSO variability due to climate change can increase TNA SST variability and the frequency of extreme TNA events^37^.

Several empirical modelling studies showed that reliable precipitation forecasts for northeastern Brazil are achieved using climate information from the tropical Pacific and Atlantic oceans. For instance, a Maximum Covariance Analysis (MCA) was applied to May–July Pacific and Atlantic SST anomalies to predict South American rainfall anomalies of the following November-January^38^. This model presented comparable probabilistic skill with a dynamical multi-model ensemble in the north of northeastern Brazil. Furthermore, a stepwise multiple regression based on October-January precipitation data from northeast Brazil rainfall monitoring stations and January SST and wind indices from the Pacific and Atlantic was used to predict March-June rainfall in the region^25^. They concluded that the empirical model produced forecasts with smaller errors and bias than ECHAM4.5 postprocessed with model output statistics (MOS) methods.

Recently, a linear regression was employed to model the relationship between lagged global SST anomalies with the leading February-April (FMA) spatial precipitation modes in northeast Brazil derived from a Principal Component Analysis (PCA)^39^. The regression model was used to predict the leading PCA modes that were then transformed back to FMA precipitation and converted to probabilistic forecasts. The authors reported that the empirical model was better calibrated for the below-normal category than the NMME, whereas the reverse was true for the above-normal category.

A comprehensive evaluation of the ensemble learning approach for hydroclimate forecasting still constitutes a gap in this research field. Therefore, the present study aims to fill that gap by comprehensively evaluating an EANN model's deterministic and probabilistic seasonal precipitation forecasting skill. The study also emphasizes the differences between EANN, traditional statistical and state-of-the-art dynamical models. In order to accomplish this, we evaluate the forecasting skill of a 1-month-lead EANN based on large-scale climate oscillation indices to forecast the FMA precipitation spatial distribution in the Ceará state, northeastern Brazil. The term “month-lead” refers to the time difference in months between forecast time issuance and forecast time validity^40^. The EANN performance is compared to traditional statistical and dynamical models that constitute Ceará’s operational seasonal forecasting system. Moreover, the advantages of combining ensemble learning and dynamical models into a hybrid MME are explored.

We chose the Ceará state because it is inserted in one of the most predictable regions on the planet in terms of seasonal forecasting^24,25^, where both empirical and dynamical models have overall good seasonal forecasting skills. Moreover, the analysis is carried out in the FMA season due to its high interannual variability (Supplementary Fig. 1). Finally, the Ceará Foundation for Meteorology and Water Resources (Funceme) provides a daily precipitation gridded data set based on the interpolation of its dense rainfall monitoring network. This network has been subject to several studies^25,41–47^.

The remainder of the paper is organized as follows: Sect. "Data" presents the data sets. Section "Methods" describes the methodology used to construct the EANN and the verification procedure. The comparison between statistical and dynamical models is discussed in section "Results", followed by an evaluation of possible MME combinations. Finally, Section "Summary and discussion" presents a summary, a discussion of the main results, and recommendations for future empirical modelling studies.

## Data

### Gridded daily precipitation data

The Funceme’s daily precipitation data set is a gridded data set with a spatial resolution of 0.15° × 0.15°, which spans from 1974 to the present. It is constructed based on an ordinary kriging interpolation of the 550 non-recording rain gauges that cover all 184 municipalities of Ceará. When transmitted to the institute, the observations go through an internal consistency check, and prior to the interpolation, they are submitted to an outlier’s filter (personal communication, 2022). This gridded data set is updated daily and is one of the main monitoring tools used by the institute to assess the temporal and spatial distribution of precipitation over several timescales^48^.

The following will briefly describe the stations' geographical distribution and density per grid cell (Supplementary Fig. 2). A detailed evaluation of the Funceme gridded data set is beyond the scope of this paper. In the first decade, the coverage was coarse, and most stations were in the northern coast, northwestern, and southern parts of Ceará (Supplementary Fig. 2a)—only 26% of the grid cells comprised at least one station during this period. Through the 1980s (Supplementary Fig. 2b and g) and 1990s (Supplementary Fig. 2c and h), the number of rain gauges increased across all regions of Ceará, and the number of grid cells with at least one station grew to 37% and 44%, respectively. The period between 2000 and 2009 depicted the network's most significant expansion, and the rain gauge spatial distribution became more homogeneous (Supplementary Fig. 2d). The station density also considerably improved, and the number of grid cells comprising at least one station increased to 55% (Supplementary Fig. 2i). In the last decade, the stations’ geographical distribution and density remained unchanged (Supplementary Figs. 2e and j).

### Explanatory and response variables

The response variable of the present study is the FMA total precipitation over the Ceará state, computed at each grid point of Funceme’s daily gridded data set. As explanatory variables, October–November-December (OND) averaged values of the Oceanic Niño Index (ONI), TNA and TSA indices are used. The Extended Reconstructed SST v5^49^ and 10 m wind from ERA5 reanalysis^50^ are used to compute the indices. The linear trend is removed from all gridded data sets at each grid point before the analysis.

The ONI is computed as a 3-month running mean of the SST anomalies in the Niño 3.4 region. Both Atlantic indices are derived from an MCA applied to SST and 10-m wind anomalies over the tropical Atlantic^30^. In the original paper, the MCA applied to SST and 10 m wind between 1948 and 2001 depicts the Atlantic Meridional Mode^51^ as the leading mode. In the present study, an MCA applied to the period between 1982 and 2015 reveals the TNA and TSA as the first and second modes, respectively. Therefore, the TNA and TSA indices are constructed by projecting the first and second pattern coefficients onto the SST anomalies. When applied to the period between 1952 and 2001, we obtained similar results to the original paper.

Maps of Spearman correlation are used to measure the monotonic relationship between OND climate indices and FMA precipitation anomalies at each grid point. The Spearman correlation is simply the Pearson correlation computed using the ranks of data, which can be simplified to

1 $$r=1-\frac{6\sum_{i=1}^{n}{D}_{i}^{2}}{n\left({n}^{2}-1\right)},$$

where D_i_ is the difference in ranks between the *ith* of n data pairs.

### Dynamical models forecasts

The NMME is a coupled ocean–atmosphere forecast system that produces real-time forecasts on the seasonal-to-interannual time scales since August 2011. The ensemble comprises coupled dynamical models from several institutions in the United States and Canada. We use 1982–2021 January initializations of the February, March and April forecasts of monthly precipitation rates from three models that constitute the latest version of the NMME (NMME4) project^52^. These values are converted to FMA total precipitation. The other four models that constitute the NMME4 are not used because the 2021 January initializations were not available for download by the time the analysis was conducted.

We also use the FMA total precipitation forecasts issued in January from the ECHAM4.6 model, an Atmospheric Global Circulation Model developed at Max Planck Institute for Meteorology and configured at T42 spectral truncation, giving a spatial resolution of approximately 2.8°, and with 19 vertical levels from the surface to 10 hPa. A 20-member ECHAM4.6 ensemble was operationally implemented at Funceme’s data center to produce real-time seasonal forecasts in 2011. An AMIP-type run models the initial conditions of the atmosphere (starting in 1961), and the model is forced by persisted monthly observed SSTs (NOAA Optimum Interpolation SST V2)^53^.

The outputs of each dynamical model are bilinearly interpolated onto Funceme’s precipitation data set grid resolution for forecast verification. The relevant information about the models used in this study is shown in Supplementary Table 1.

## Methods

### Ensemble of artificial neural networks

Machine learning models such as ANN can learn complex nonlinear relationships between explanatory and response variables. ANN is one of the most frequently used nonlinear regression methods since it can approximate every sufficiently smooth function of the inputs, yielding low-bias estimates^19^. On the other hand, ANNs are considered unstable predictors because they are sensitive to small changes, for instance, in their topology, initial weights and training set^54^. Changing one or several of these aspects results in a different network with different generalization patterns (high variance). A successful way to address this problem is combining multiple networks with small changes among them to accomplish the same task^21,55,56^. Each ensemble member is known to make different errors, but when combined, their similarities (signal) are highlighted, whereas their differences (noise) are diminished^18^.

This study employs the multi-layer perceptron ANN, a feedforward network consisting of three layers: input, hidden and output. Each ANN is trained using the standard backpropagation algorithm^57^, which updates the weight matrix using the gradient of the loss function and a learning rate parameter set as 10^–1^. The ANN architecture comprises one input layer with a number of units equal to the number of explanatory variables, one hidden layer with three units and one output layer with one unit. L2 regularization is used to reduce overfitting, which inflates the loss function by adding the squared magnitude of coefficients multiplied by a regularization constant set as 10^–3^. The hyperbolic tangent and linear activation functions are used in the hidden and output layers, respectively. The network training stops when the gradient of the loss function is less than 10^–2^ or reaches up to 10,000 epochs. The models are implemented using Tensorflow and Keras libraries for Python 3.9.

The ensemble members are derived using the Bagging algorithm, an approach based on the bootstrap statistical resampling to create diverse subsets from the original training set^58^. The subsets have the same size as the original training set and are created by random sampling with replacement of the n instances. Each instance has a probability 1/n of being chosen to populate a subsample.

**Figure 1:**
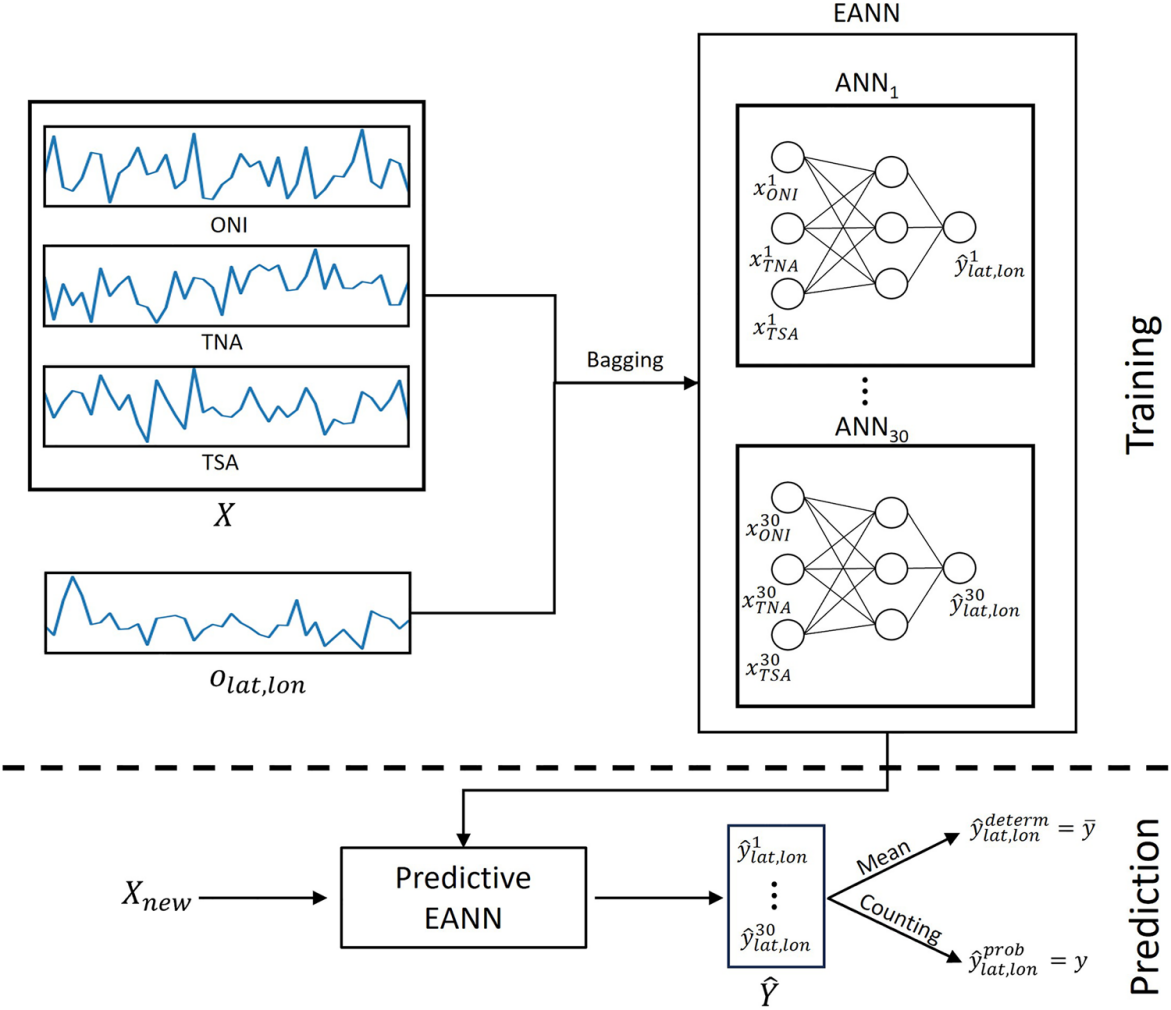
Flowchart illustrating the training and prediction procedures. In the training phase, the training sample is resampled with replacement to create 30 sub-samples. Subsequently, each sub-sample is used to train an ANN. All ANNs use the same hyperparameters. In the prediction phase, a new sample goes in the trained ANNs, generating 30 different predictions. The predictions are combined through mean and counting methods, respectively, resulting in a deterministic and probabilistic forecast. The indices lat and lon represent the latitude and longitude of a specific grid point, indicating a point-wise training and prediction process.

An ANN ensemble is trained at each precipitation data set grid point. The number of ensemble members is set to 30. As shown in the results (subsection "Effect of the ensemble size"), this number is enough to achieve good generalization ability. The same ANN hyperparameters are used in every grid point and were defined through trial and error. Specifically, the leave-one-out cross-validation (described in subsection "Forecast verification and evaluation metrics") is conducted for different combinations of hyperparameters. The combination that gives the best cross-validated RMSE results is shown in this paper. A flowchart illustrating the training and prediction procedures is shown in Fig. 1.

### Traditional statistical models

A multiple linear regression (MLR) based on the ordinary least squares algorithm is implemented for deterministic forecasts^59^. For probabilistic forecasts, a multinomial logistic regression (MNLR) is implemented^60^. This extension of the binary logistic regression supports multi-class classification problems. The MNLR parameters are optimized by maximizing the log-likelihood function.

### Forecast verification and evaluation metrics

The evaluation of each ensemble is conducted through leave-one-out cross-validation. This procedure uses all observations of the predictand to estimate the prediction errors in a way that allows each observation to be treated, one at a time, as independent data^61^.

For each dynamical model, we employ leave-one-out cross-validation between 1982 and 2021 to compute standardized anomalies. The held-out year is subtracted from the model's long-term mean and then divided by its long-term standard deviation, both computed on the remaining 39 years. The evaluation metrics are then computed on each held-out standardized anomaly and then averaged. Using the model's long-term mean and standard deviation when computing the anomalies corrects for both systematic bias in the mean and spread of the model^62^.

For the EANN, each year between 1982 and 2021 is left out, and the long-term mean and standard deviation are computed on the remaining 39 years. Subsequently, the standardized anomalies are computed for those 39 years and the years between 1975 and 1981, yielding 46 training samples. The period between 1982 and 2021 was used to compute the long-term statistics for consistency with the computation of the dynamical model standardized anomalies. Moreover, the anomalies for the held-out year are also computed using the same long-term statistics as the training set. Following, the training samples are resampled 30 times with Bagging, and a model is fitted for each sub-sample. The fitted models are used to predict the omitted observation, yielding 30 predictions. Finally, these predictions are combined through a simple mean and counting method (described below), and the evaluation metrics are computed between the final predicted value and the omitted observation. The training and prediction procedures are illustrated in Fig. 1. This process is repeated for each held-out year, resulting in 40 independent error values, and the model's true performance is computed by averaging those errors.

Deterministic forecasts are formed by simple ensemble mean. Probabilistic forecasts are formed by counting the number of members that fall in each of the equiprobable categories above normal (AN), near normal (NN) and below normal (BN) and dividing by the total number of members. We assume that FMA precipitation anomalies in the Ceará state follow a Gaussian distribution. Thus, standardized anomalies above + 0.43 are considered AN, between + 0.43 and − 0.43 are considered NN and below − 0.43 are considered BN. This is a reasonable assumption since the Yule-Kendall skewness index for FMA precipitation is near zero in this region^39^.

The deterministic evaluation metrics used are the Bias, which expresses the mean error of the forecasts, and the Root Mean Squared Error (RMSE), which measures the accuracy of the forecasts

2 $$Bias=\frac{1}{n}\sum_{i=1}^{n}\left({\overline{y} }_{i}-{o}_{i}\right) ,$$

3 $$RMSE=\sqrt{\frac{1}{n}\sum_{i=1}^{n}{({\overline{y} }_{i}-{o}_{i})}^{2}} .$$

where (${\overline{y} }_{i}$, ${o}_{i}$) are the *ith* of the n pairs of ensemble average and observation.

The probabilistic performance is measured through the Ranked Probability Score (RPS), which is an evaluation metric for multicategory events defined as

4 $$RPS= \frac{1}{n}\sum_{i=1}^{n}\sum_{\mathrm{m}=1}^{J}{\left[\left(\sum_{j=1}^{m}{y}_{i,j}\right)-\left(\sum_{j=1}^{m}{o}_{i,j}\right)\right]}^{2} ,$$

where y_i,j_ and o_i,j_ are the *ith* of the n forecast and observation pairs for the *jth* category of the J categories.

Reliability and sharpness diagrams are used to assess three important aspects of probabilistic forecasts: reliability, resolution and sharpness. Reliability measures the consistency between the forecast probabilities and the relative frequency of the observed outcomes. Resolution quantifies the degree to which the observed outcomes change as the forecasts change. Sharpness expresses how often each forecast probability is issued^63^. This study's reliability and sharpness diagrams are based on a binning of K = 10 forecast probabilities over the whole geographic domain (area aggregated). Reliability and resolution of probabilistic forecasts can also be described as scalars by decomposing the Brier score^64^:

5 $$REL=\frac{1}{N}\sum_{k=1}^{K}{N}_{k}{\left({y}_{k}-{\overline{o} }_{k}\right)}^{2} ,$$

6 $$RES=\frac{1}{N}\sum_{k=1}^{K}{N}_{k}{\left({\overline{o} }_{k}- \overline{o }\right)}^{2} ,$$

where N_k_ is the number of times each forecast y_k_ is used in the collection of K forecasts being verified, with $N={\sum }_{k=1}^{K}{N}_{k}$. The conditional average observation ${\overline{o} }_{k}$, is expressed as

7 $${\overline{o} }_{k}=\frac{1}{{N}_{k}}\sum_{l\in {N}_{k}}{o}_{l} ,$$

where ${o}_{l}=1$ if the event occurs for the *lth* forecast-event pair, ${o}_{l}=0$ otherwise, and the summation is over only those values of l corresponding to occasions when the forecast y_k_ was issued. The sample climatology, $\overline{o }$, is given by

8 $$\overline{o }=\frac{1}{N}\sum_{k=1}^{K}{N}_{k}{\overline{o} }_{k} .$$

The reliability and resolution terms are negatively and positively oriented, respectively.

Confidence intervals for the reliability diagram statistics, reliability and resolution terms are determined using bootstrapping^65^. The forecast-observation grid point pairs are resampled 1000 times, and the statistics are computed for each resulting sampling. The 2.5th and 97.5th percentiles determine the confidence intervals.

## Results

### Effect of the ensemble size

This section explores the effect of the ensemble size on the errors’ spatial distribution. Supplementary Fig. 3 shows boxplots summarizing the changes of cross-validation Bias (top panel), RMSE (middle panel), and RPS (bottom panel) over all the grid points with respect to the ensemble size.

Both Bias (Supplementary Fig. 3—top panel) minimum and maximum values are almost reduced by half as the number of members increases from 1 to 5. They are further reduced and then stabilized with a 30-member ensemble. An interesting aspect of the bias distribution is that the median does not change, suggesting that the single model is enough to produce low-bias estimates in some grid points. Nevertheless, increasing the ensemble size results in a bias reduction in most of the grid points, evidenced by the narrowing of the distribution.

For the RMSE (Supplementary Fig. 3—middle panel) and RPS (Supplementary Fig. 3—bottom panel), not only do the distributions become narrower with the increase of the ensemble size but the median is also reduced, suggesting that there is an overall improvement in the generalization ability. As in the bias case, stability is achieved with an ensemble of 30 members.

### Comparison of empirical and dynamical models

#### Deterministic evaluation of individual models

**Figure 2:**
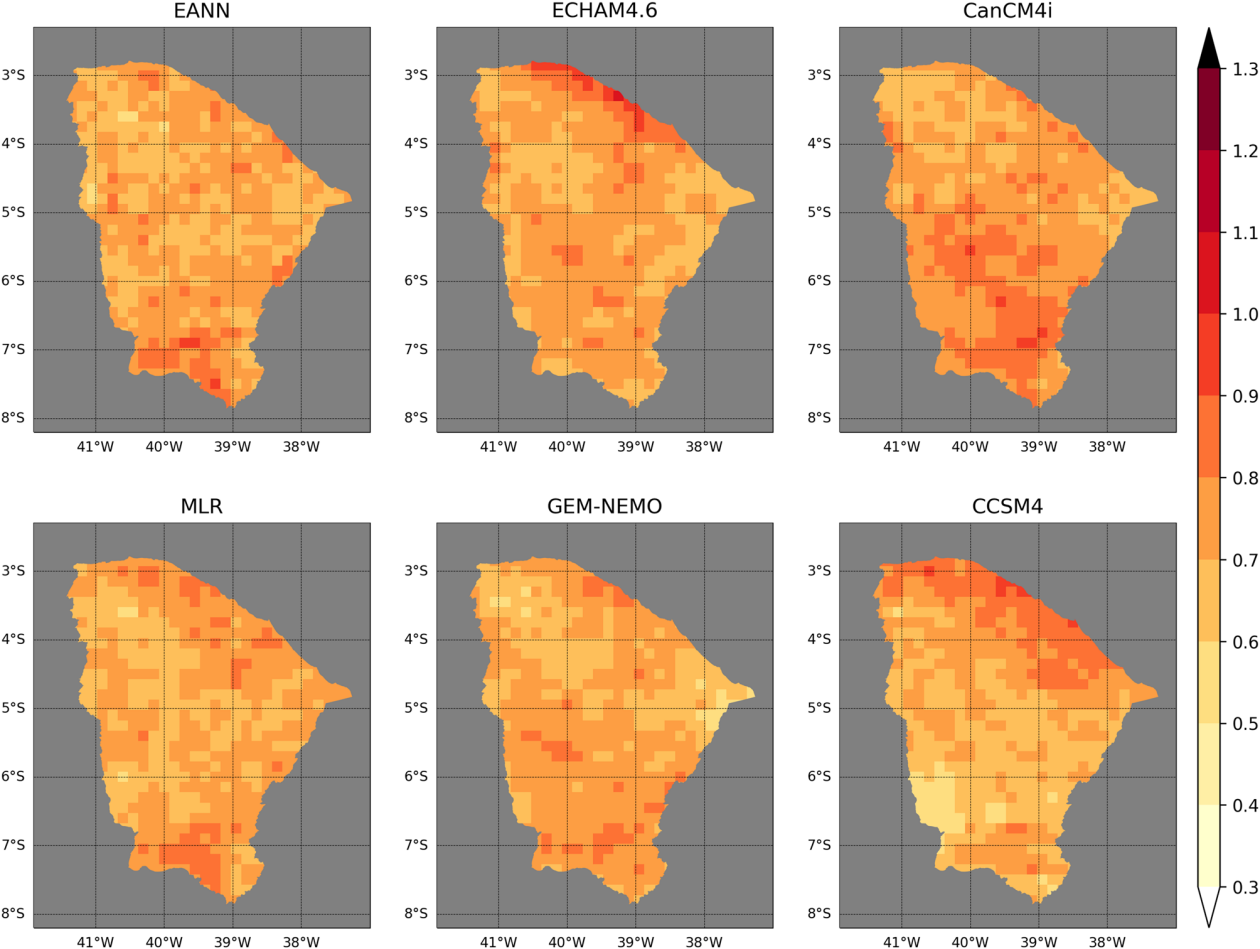
Cross-validation RMSE maps of the EANN (top left), MLR (bottom left), ECHAM4.6 (top middle), GEM-NEMO (bottom middle), CanCM4i (top right) and CCSM4 (bottom right). The colorbar is in standardized units.

The deterministic accuracy, measured as RMSE, is shown in Fig. 2. The statistical models performance in the northern Ceará resembles the best dynamical models, i.e., the CanCM4i and the GEM-NEMO. The EANN has better accuracy than the MLR close to the coast. The TSA index has the highest Spearman correlation with FMA precipitation (Supplementary Fig. 4) in this region. Other studies also confirm that lagged SST anomalies in the southern tropical Atlantic have good correlations with FMA precipitation anomalies in northern Ceará^39,66,67^.

In the western Ceará, all three indices have important signals, although each has its highest correlation values in different areas. TSA is the most important index in the northern area, close to the coast, with correlations above 0.4 in most grid points, followed by ONI. TNA does not play an important role there. All models present similar error patterns in this area, except CCSM4. ONI is the most important predictor in the central-western region, followed by TSA. TNA correlations increase, ranging between -0.2 to -0.4 in most of this area. All models perform similarly, although ECHAM4.6 and CCSM4 have slightly better performance. Further south, TNA is the index with the highest correlation values, followed by ONI and TSA. In this region, CCSM4 is the model with the lowest RMSE, whereas CanCM4i and GEM-NEMO present the highest error values.

In the eastern and central regions of Ceará, the indices have smaller correlations with precipitation than in the western and northern parts. ONI and TSA have absolute correlation values ranging from 0.2 to 0.4 in most grid points, while TNA correlations are between − 0.2 and 0 overall. ECHAM4.6 and GEM-NEMO are the two models with the smallest errors in the northeastern region, whereas CCSM4 and the statistical models perform better in the central region.

In the southern Ceará, both Atlantic indices have absolute correlation coefficients below 0.2, and most of the diminished skill comes from ONI. This is a high-altitude region and far from the Atlantic. Although the Atlantic ITCZ mainly controls the rainfall regime there, orography and the influence of frontal systems also play important roles^68^, which could explain the small correlation coefficients. The limited climate indices signal results in the worst performance of the statistical models in terms of RMSE, except for the eastern boundary, where ONI presents moderate correlations (− 0.6 to − 0.4) with precipitation. CCSM4 and ECHAM4.6 have the highest accuracy in this region, and CanCM4i has the lowest, followed by the EANN and the MLR.

A summary of the deterministic accuracy is shown in Table 1. CCSM4 has the lowest median RMSE, followed by the statistical models. CanCM4i has the highest median error. A two-sample *t*-test at a 5% significance level is performed to assess the null hypothesis of equal population mean among models’ forecasts (Supplementary Table 2). The EANN is statistically different from three out of the five models. ECHAM4.6 and GEM-NEMO are statistically different from every other model, whereas CanCM4i and the MLR are only different from two of the other models.

**Table 1:** A summary of the median metrics of the field forecasts.

Model	Metric	
	RMSE	RPS
EANN	0.71	0.38
ECHAM4.6	0.72	0.40
CanCM4i	0.75	0.40
MLR/MNLR	0.71	0.39
GEM-NEMO	0.72	0.37
CCSM4	0.70	0.41

These metrics are computed over each grid point and averaged over time.

#### Probabilistic evaluation of individual models

**Figure 3:**
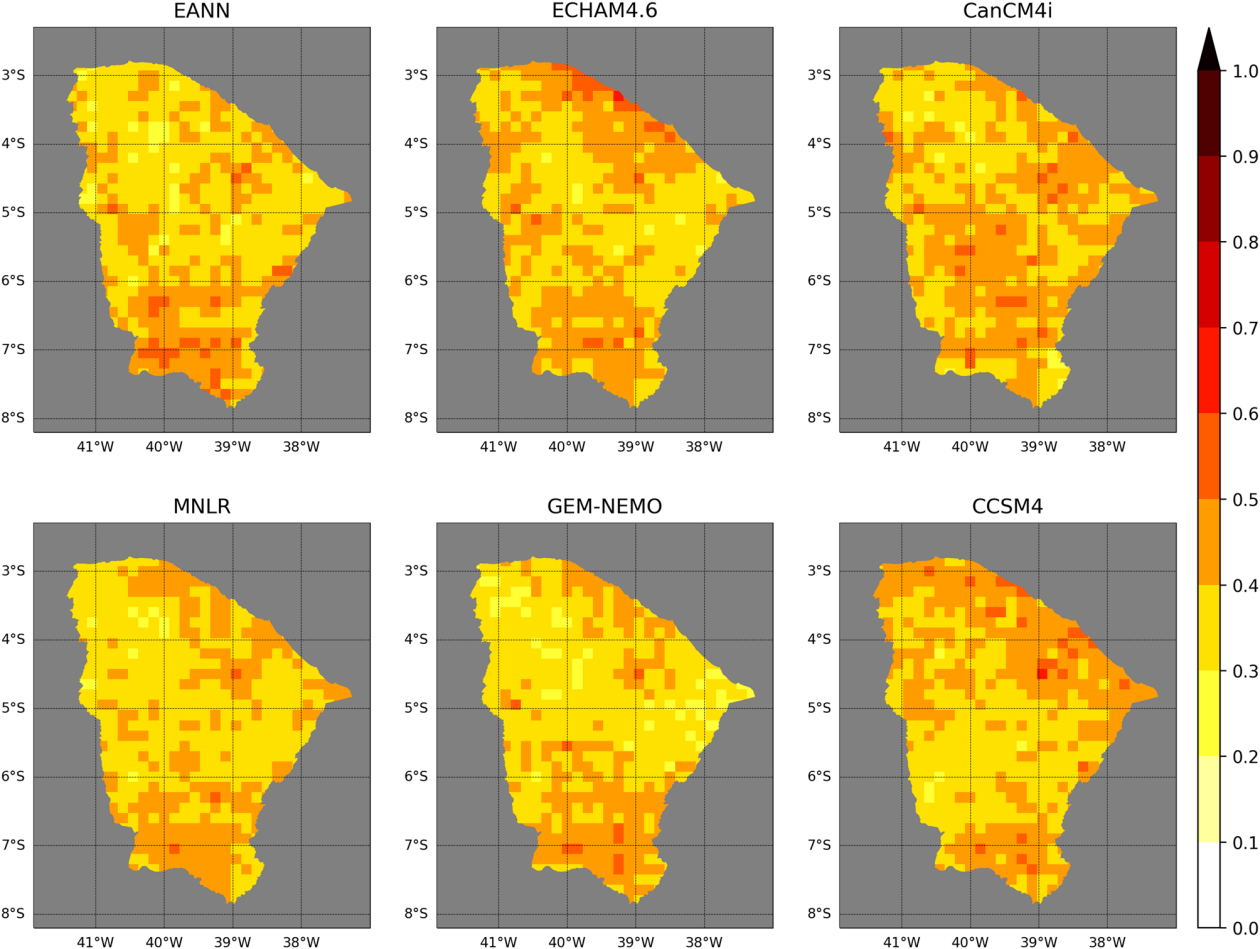
Same as Fig. 2 but for RPS.

The maps of cross-validation RPS (Fig. 3) resemble those of RMSE. Overall, the statistical models have more accuracy in the central-northern region of Ceará than most of the dynamical models (northward of 5°S). Nevertheless, GEM-NEMO outperforms all other models in this region, except for the northern border, where the EANN stands out. CCSM4 has the best accuracy between 5° and 7°S, reproducing the RMSE pattern. All models present high RPS in the southmost region, with the EANN having overall higher values than the other models.

**Figure 4:**
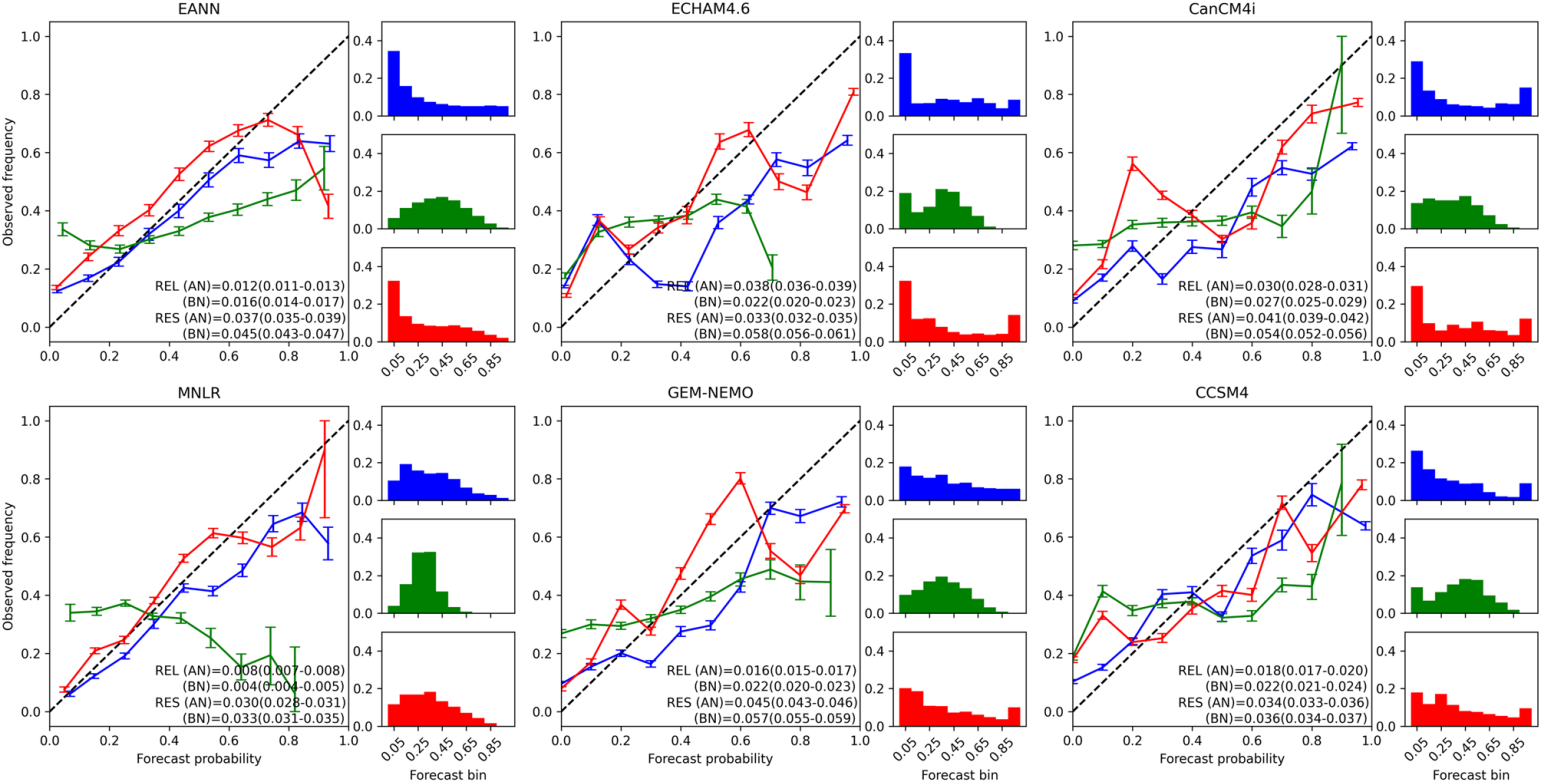
Reliability and sharpness diagrams of the EANN (left), ECHAM4.6 (top middle), GEM-NEMO (bottom middle), CanCM4i (top right) and CCSM4 (bottom right). Blue lines and bars represent forecasts in the above tercile, green the normal and red the below. Alphanumeric insets show the reliability (REL) and resolution (RES) terms of the Brier Score. Error bars and values in parenthesis indicate 95% bootstrap confidence intervals.

The reliability and sharpness diagrams, along with the reliability (REL) and resolution (RES) terms for each equiprobable category, are shown in Fig. 4. The statistical models provide the best-calibrated probabilistic forecasts for BN and AN categories, supported by the smallest area-aggregated reliability values. When comparing statistical models, the MNLR has a lower REL, while the EANN has a better RES. This difference can be understood by examining their sharpness diagrams. Most of the MNLR probability density is close to the climatological probability (0.33), resulting in a low-resolution term (bars in the bottom-left panel of Fig. 4). On the other hand, the EANN sharpness diagram (bars in the top-left panel of Fig. 4) shows that the extreme probabilities are issued more often than the climatological probability. For instance, the AN largest probabilities (0.9–1.0) are issued almost as many times as the probability range containing the climatology (0.3–0.4).

The EANN reliability diagram reveals a good agreement between forecast probabilities and their relative observed frequencies for forecast bins between 0 and 0.8 of the BN category, although with small under-forecasting biases (red line in Fig. 4—top-left panel). The AN category is also well-calibrated for forecast bins between 0 and 0.7 (blue line in Fig. 4—top-left panel). However, for large forecast probabilities (> 70% for AN and > 80% for BN), the EANN presents over-forecasting biases. The sharpness diagram reveals that the model often used the BN and AN smallest probabilities (0–0.1), which is expected since in the presence of a strong signal (a strong El Nino or La Nina, for instance), the model members usually agree that one of the extreme categories has a low likelihood of occurrence^69^. Nevertheless, because of the high variance of ANN models, there is hardly a general agreement among all members that the opposite tercile is the most likely one, with some falling in the NN category. This is supported by the low frequency of the largest probability bin (0.9–1.0) of both extreme terciles and the smallest probability bin (0–0.1) of the NN tercile in the EANN sharpness diagram (Fig. 4—top-left panel).

The dynamical models have calibration-function slopes shallower than the 1:1 reference line for BN and AN categories, indicating overconfident forecasts. CCSM4 (Fig. 4—bottom-right panel) and GEM-NEMO (Fig. 4—bottom-middle panel) provide the best-calibrated probabilities for BN (red line) and AN (blue line) categories among the dynamical models, evidenced by their low REL term. GEM-NEMO features higher RES terms of the extreme categories than the other models (except for the BN category of ECHAM4.6), indicating good discerning between different observed situations. ECHAM4.6 presents over-forecasting biases associated with probabilities above 0.3 of the AN category (blue line in Fig. 4—top-middle panel). CanCM4i and GEM-NEMO have similar biases, although to a lesser degree than ECHAM4.6. All dynamical models often use the largest and smallest probabilities of BN and AN categories, consistent with overconfidence. Overall, they all have better resolution than the EANN.

Both empirical and dynamical models depict a bad-calibrated NN category with poor resolution. This is a well-known deficiency of seasonal forecasting systems since strong signals do not substantially influence probabilities in the NN category as in the extreme categories and, thus, are less likely to fall beyond the climatology forecast^69,70^.

A summary of the probabilistic accuracy is shown in Table 1. GEM-NEMO has the lowest median RPS, followed by the EANN. CCSM4 has the highest median RPS.

### Forecast verification of multi-model ensembles

**Figure 5:**
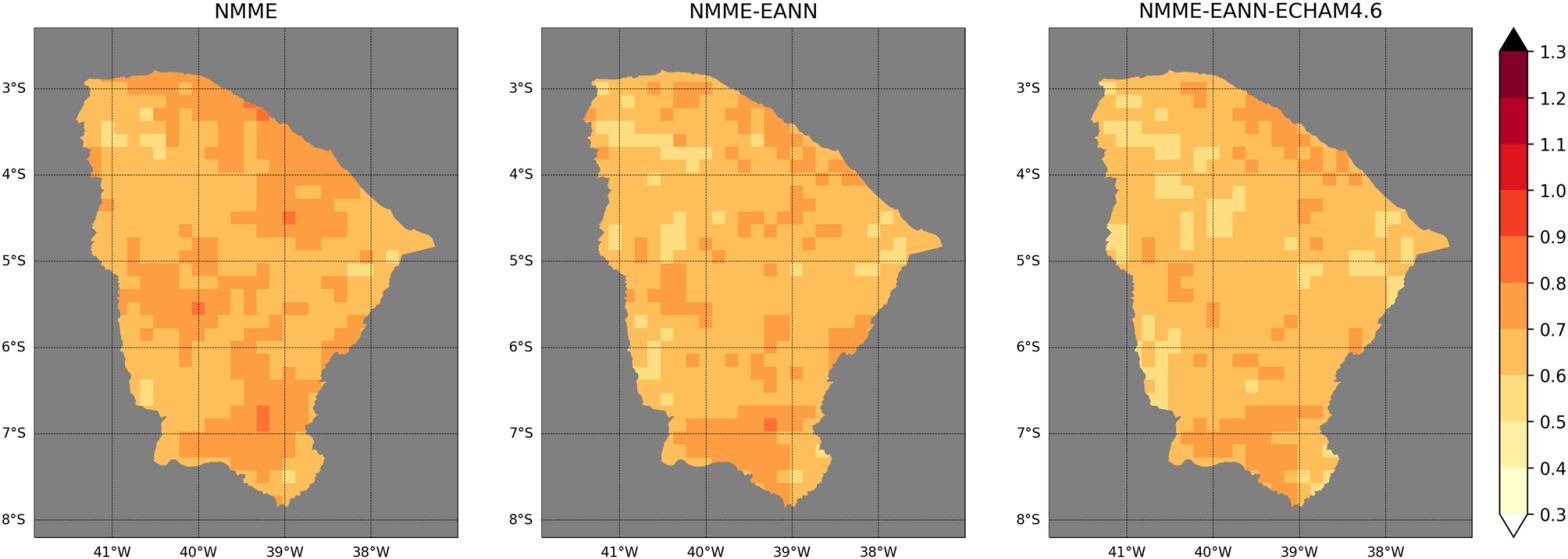
Cross-validation RMSE maps of the MME combinations made of: NMME models (left), NMME models and EANN (middle) and NMME models, EANN and ECHAM4.6 (right). The colorbar is in standardized units.

**Figure 6:**
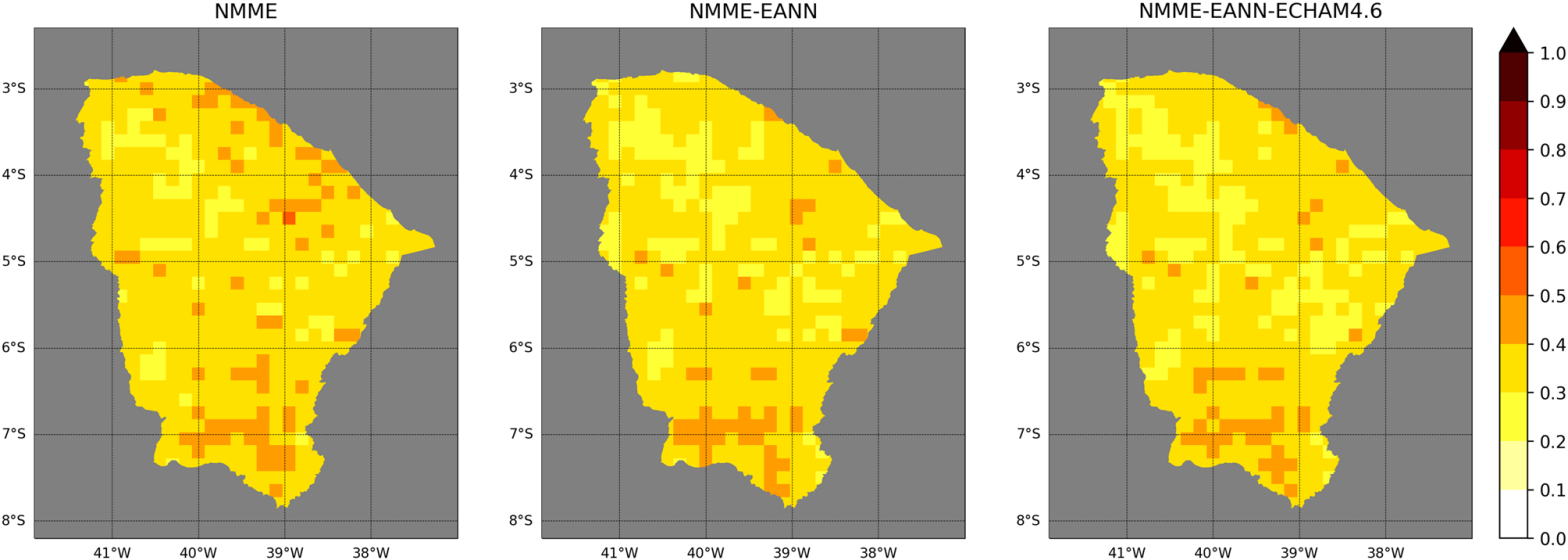
Same as Fig. 5 but for RPS.

**Figure 7:**
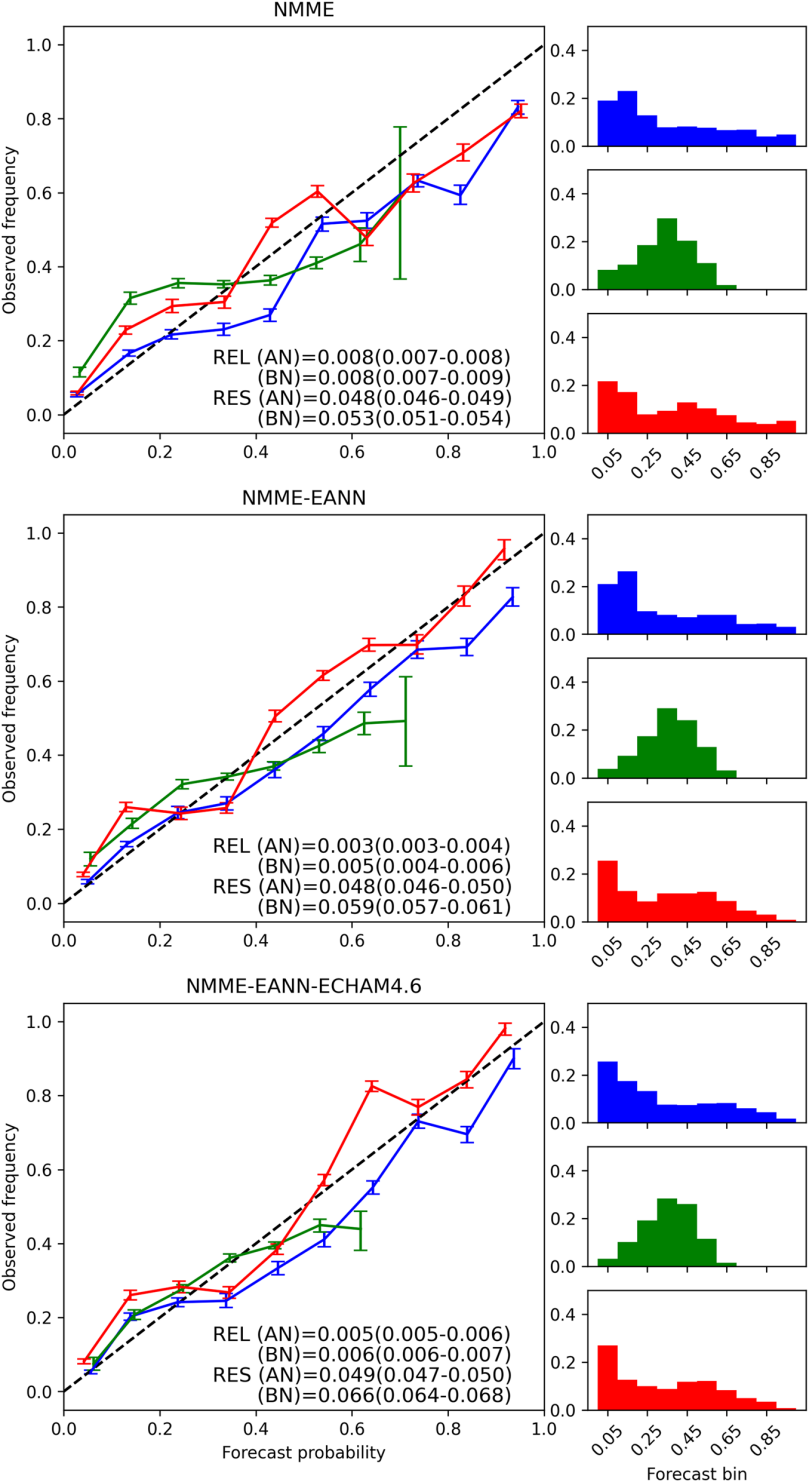
Same as Fig. 4 but for the MMEs made of: NMME models (top), NMME models and EANN (middle) and NMME models, EANN and ECHAM4.6 (bottom).

The MME made of the individual NMME models results in an overall reduction of the RMSE (Fig. 5—left map) and RPS (Fig. 6—left map). Consequently, RES and REL terms of all equiprobable categories also improve (Fig. 7—top panel). The most striking impact of using the MME is the reduction of conditional biases, evidenced by calibration functions (lines in Fig. 7—top panel) that deviate less from the reference 1:1 line than the individual models.

Nevertheless, using only dynamical models in the MME still results in overconfident forecasts, depicted by calibration-function slopes shallower than the reference 1:1 line. Calibration of overconfident forecasts relies on adjusting the extreme probabilities to be less extreme^61^. Including the EANN in the MME improves this aspect by inhibiting the excessive use of the largest probabilities. This can be observed by a reduction of the last forecast bin on the sharpness diagram of both AN (blue bars) and BN (red bars) categories when the EANN is combined with the NMME models (Fig. 7—middle panel). This reduction is more pronounced in the BN than in the AN category, which is explained by the higher frequency of the latter category than the former in the EANN sharpness diagram. Both REL and RES terms are improved. Another study also found that combining an MCA forecasting model and dynamical models from the DEMETER project improved the reliability and resolution of seasonal rainfall forecasts in northeastern Brazil compared to individual predictions^38^. Moreover, including the EANN in the MME leads to an overall reduction of RMSE (Table 2—middle column) and RPS (Table 2—right column), especially in the central and northern areas (Fig. 5 and Fig. 6—middle maps).

**Table 2:** Same as Table 1 but for the MME combinations.

Model	Metric	
	RMSE	RPS
NMME	0.69	0.35
NMME-EANN	0.66	0.34
NMME-EANN-ECHAM4.6	0.54	0.33

Incorporating ECHAM4.6 into the hybrid MME further reduces the RMSE (Fig. 5—right map, Table 2—middle column) and RPS (Fig. 6—right panel, Table 2—right column). Nevertheless, a degradation of the REL term is observed due to an increase of middle-range probabilities over-forecasting bias of both BN (red line) and AN (blue line) categories (Fig. 7—bottom panel).

As in the case of the individual models, a two-sample *t*-test is performed to check whether the MMEs forecasts are different in the population mean (Supplementary Table 3). Only the NMME-EANN and the NMME-EANN-ECHAM4.6 are statistically different.

## Summary and discussion

This study assessed the deterministic and probabilistic performance of a 1-month-lead EANN using OND climate indices from the Atlantic and Pacific Oceans to forecast the FMA precipitation anomalies in Ceará, northeast Brazil. We also proposed integrating the forecasts of the EANN and dynamical models and analyzed the advantages of using this hybrid MME.

The EANN deterministic and probabilistic performance closely followed the lagged correlation between climate indices and precipitation. Its performance is better in regions where at least one index has moderate correlation coefficients (e.g., northern Ceará) or where multiple indices are less correlated with FMA precipitation (e.g., eastern Ceará). On the other hand, the model’s worst performance is observed in the southern region, where only ONI has a weak signal. A spatial comparison of the EANN with traditional statistical models and the dynamical models that currently constitute the operational seasonal forecasting system of Ceará showed that the EANN was among the models with the smallest RMSE and RPS in most regions.

The analysis of area-aggregated probabilistic statistics showed that the EANN is a well-calibrated model with intermediate confidence. Its sharpness diagrams revealed that it issues fewer probability forecasts close to the climatology than the MNLR, resulting in its better resolution but worse reliability. On the other hand, the EANN issues fewer large probabilities than dynamical models, resulting in a worse resolution but a better calibration of the former. Further analysis of the EANN sharpness diagram indicated underconfidence in issuing the largest probabilities (0.9–1.0) of AN and BN categories due to its high inter-member variance that hindered a general agreement among all single networks. Good forecasting requires both reliability and resolution, but neither attribute alone is sufficient. Therefore, achieving a balance between them is a favorable characteristic of the EANN.

The MME composed of NMME models improved the deterministic and probabilistic forecasting skills across all regions of Ceará compared to the results of individual models. It also led to better-calibrated forecasts. Nevertheless, the MME composed only of dynamical models yielded overconfident forecasts. Integrating the EANN improved this aspect by preventing the excessive use of the highest probabilities of both BN and AN categories, enhancing reliability and resolution terms. An overall reduction of RMSE and RPS was also observed, especially in the central and northern regions. Furthermore, adding ECHAM4.6 to the hybrid MME further improved forecasting skills. However, the extreme categories area-aggregated reliability was degraded due to an increase of middle-range probabilities over-forecasting bias.

According to these results, the EANN is a powerful seasonal forecasting tool with different forecasting characteristics from traditional statistical and dynamical models. In addition, the EANN is being easy to implement and computationally cheaper than dynamical models. Moreover, we also show that integrating ensemble learning and dynamical models into a hybrid MME leads to better probabilistic forecasts. This result encourages further research and application of such hybrid forecasting systems.

Further steps include evaluating the seasonal forecasting skill of the EANN for longer lead times and other regions of the globe and improving aspects of the modelling procedure. For instance, an MOS method could replace the nonparametric count method for better-calibrated probabilistic forecasts^71^. Moreover, the predictors used were indices that require prior knowledge of the climate modes that impact the regional climate variability and exhaustive testing of potential methods to compute those indices. Therefore, improvements could be achieved using a more generalized input variable selection method. For instance, a recent study defined the predictors as a linear combination of global temperature field (SST over ocean and 2-m air temperature over land)^72^. In this method, the point-wise correlation of the temperature field and the predictand worked as weights for the linear combination.

### Supplementary Information

Supplementary Information.

## Data Availability

The datasets generated and/or analysed during the current study are available in the Funceme, NOAA and Copernicus repositories, http://www3.funceme.br/web/storage/obs/interpolation_kriging_funceme_valid_rain/, https://www.ncei.noaa.gov/products/extended-reconstructed-sst, https://cds.climate.copernicus.eu/cdsapp#!/dataset/reanalysis-era5-single-levels-monthly-means?tab=overview.

## References

[1] Kushnir Y, Robinson WA, Chang P, Robertson AW (2006). The physical basis for predicting Atlantic sector seasonal-to-interannual climate variability. J. Clim..

[2] Trenberth KE (1997). The definition of El Niño. Bull. Am. Meteorol. Soc..

[3] Shabbar A, Bonsal B, Khandekar M (1997). Canadian precipitation patterns associated with the Southern Oscillation. J. Clim..

[4] Kumar KN, Ouarda TBMJ (2014). Precipitation variability over UAE and global SST teleconnections. J. Geophys. Res. Atmos..

[5] Chandran A, Basha G, Ouarda TBMJ (2016). Influence of climate oscillations on temperature and precipitation over the United Arab Emirates. Int. J.Climatol..

[6] Ouachani R, Bargaoui Z, Ouarda T (2013). Power of teleconnection patterns on precipitation and streamflow variability of upper Medjerda Basin. International Journal of Climatology.

[7] Coelho CAS, Uvo CB, Ambrizzi T (2002). Exploring the impacts of the tropical Pacific SST on the precipitation patterns over South America during ENSO periods. Theor. Appl. Climatol..

[8] Hurrell JW (1995). Decadal trends in the North-Atlantic oscillation—Regional temperatures and precipitation. Science.

[9] Saji NH, Yamagata T (2003). Structure of SST and surface wind variability during Indian Ocean Dipole mode events: COADS observations. J. Clim..

[10] Liu Z, Alexander M (2007). Atmospheric bride, oceanic tunnel, and global climate teleconnections. Rev. Geophys..

[11] Tremblay L, Larocque M, Anctil F, Rivard C (2011). Teleconnections and interannual variability in Canadian groundwater levels. J. Hydrol. (Amst.).

[12] Alexander MA (2002). The atmospheric bridge: The influence of ENSO teleconnections on air-sea interaction over the global oceans. J. Clim..

[13] Coulibaly P, Anctil F, Rasmussen P, Bobe B (2000). A recurrent neural networks approach using indices of low-frequency climatic variability to forecast regional annual runoff. Hydrol. Process..

[14] Mekanik F, Imteaz MA, Gato-Trinidad S, Elmahdi A (2013). Multiple regression and Artificial Neural Network for long-term rainfall forecasting using large scale climate modes. J. Hydrol. (Amst.).

[15] Abbot J, Marohasy J (2017). Application of artificial neural networks to forecasting monthly rainfall one year in advance for locations within the murray darling basin, Australia. Int. J. Sustain. Dev. Plann..

[16] Woldesellasse H, Marpu PR, Ouarda TBMJ (2020). Long-term forecasting of wind speed in the UAE using nonlinear canonical correlation analysis (NLCCA). Arab. J. Geosci..

[17] Elshaboury N, Elshourbagy M, Al-Sakkaf A, Abdelkader EM (2021). Rainfall forecasting in arid regions using an ensemble of artificial neural networks. J. Phys. Conf. Ser..

[18] Zhang C, Ma Y (2012). Ensemble Machine Learning. Ensemble Machine Learning.

[19] Sharkey A (1999). Combining Artificial Neural Nets.

[20] Ouarda TBMJ, Shu C (2009). Regional low-flow frequency analysis using single and ensemble artificial neural networks. Water Resour. Res..

[21] Shu C, Ouarda TBMJ (2007). Flood frequency analysis at ungauged sites using artificial neural networks in canonical correlation analysis physiographic space. Water Resour. Res..

[22] Jin L (2015). A nonlinear statistical ensemble model for short-range rainfall prediction. Theor. Appl. Climatol..

[23] Xu L, Chen N, Zhang X, Chen Z (2020). A data-driven multi-model ensemble for deterministic and probabilistic precipitation forecasting at seasonal scale. Clim. Dyn..

[24] Doblas-Reyes FJ, García-Serrano J, Lienert F, Biescas AP, Rodrigues LRL (2013). Seasonal climate predictability and forecasting: Status and prospects. Wiley Interdiscip. Rev. Clim. Change.

[25] Hastenrath S, Sun L, Moura AD (2009). Climate prediction for Brazil’s Nordeste by empirical and numerical modeling methods. Int. J. Climatol..

[26] Hastenrath S (1984). Interannual variability and annual cycle: Mechanisms of circulation and climate in the tropical Atlantic sector. Mon. Weather Rev..

[27] Enfield DB, Mayer DA (1997). Tropical atlantic sea surface temperature variability and its relation to El Niño-Southern Oscillation. J. Geophys. Res. Oceans.

[28] Houghton RW, Tourre YM (1992). Characteristics of low-frequency sea surface temperature fluctuations in the tropical Atlantic. J. Clim..

[29] Moura AD, Shukla J (1982). On the dynamics of droughts in Northeast Brazil: Observations, theory, and numerical experiments with a general circulation model. J. Atmos. Sci..

[30] Chiang JCH, Vimont DJ (2004). Analogous Pacific and Atlantic meridional modes of tropical atmosphere-ocean variability. J. Clim..

[31] Xie SP, Philander SGH (1994). A coupled ocean-atmosphere model of relevance to the ITCZ in the eastern Pacific. Tellus A.

[32] Amaya DJ, DeFlorio MJ, Miller AJ, Xie SP (2017). WES feedback and the Atlantic Meridional Mode: Observations and CMIP5 comparisons. Clim. Dyn..

[33] Andreoli RV (2017). The influence of different El Niño types on the South American rainfall. Int. J. Climatol..

[34] Horel JD, Wallace JM (1981). Planetary-scale phenomena associated with the Southern Oscillation. Mon. Weather Rev..

[35] Curtis S, Hastenrath S (1995). Forcing of anomalous sea surface temperature evolution in the tropical Atlantic during Pacific warm events. J. Geophys. Res..

[36] Amaya DJ, Foltz GR (2014). Impacts of canonical and Modoki El Niño on tropical Atlantic SST. J. Geophys. Res. Oceans.

[37] Yang Y (2021). Greenhouse warming intensifies north tropical Atlantic climate variability. Sci. Adv..

[38] Coelho CAS, Stephenson DB, Balmaseda M, Doblas-Reyes FJ, van Oldenborgh GJ (2006). Toward an integrated seasonal forecasting system for South America. J. Clim..

[39] da Rocha Júnior RL (2021). An empirical seasonal rainfall forecasting model for the northeast region of Brazil. Water (Switzerland).

[40] WMO. *WMO Guidance on Operational Practices for Objective Seasonal Forecasting Guidance on Operational Practices for Objective Seasonal Forecasting*. (2019).

[41] Pinheiro E, da Rocha RP, Drumond A (2020). Assessment of 20th-century reanalysis circulation patterns associated with El Niño-Southern Oscillation impacts on the tropical Atlantic and northeastern Brazil rainy season. Int. J. Climatol..

[42] de Araújo JC, Landwehr T, Alencar PHL, Paulino WD (2023). Water Management causes increment of reservoir silting and reduction of water yield in the semiarid State of Ceará, Brazil. J. South Am. Earth Sci..

[43] Rodrigues RR, Haarsma RJ, Campos EJD, Ambrizzi T (2011). The impacts of inter-El Niño variability on the tropical Atlantic and northeast Brazil climate. J. Clim..

[44] Guerreiro MJS, Maia de Andrade E, Abreu I, Lajinha T (2013). Long-term variation of precipitation indices in Ceará State, Northeast Brazil. Int. J. Climatol..

[45] Rodrigues RR, McPhaden MJ (2014). Why did the 2011–2012 La Niña cause a severe drought in the Brazilian Northeast?. Geophys. Res. Lett..

[46] Sun L, Moncunill DF, Li H, Moura AD, de Assis de Souza Filho, F. (2005). Climate downscaling over Nordeste, Brazil, using the NCEP RSM97. J. Clim..

[47] Folland CK, Colman AW, Rowell DP, Davey MK (2001). Predictability of northeast Brazil rainfall and real-time forecast skill, 1987–98. J. Clim..

[48] Funceme. Precipitation gridded data set. (2023).

[49] Huang B (2017). Extended reconstructed Sea surface temperature, Version 5 (ERSSTv5): Upgrades, validations, and intercomparisons. J. Clim..

[50] Hersbach H (2020). The ERA5 global reanalysis. Q. J. R. Meteorol. Soc..

[51] Xie SP, Carton JA (2004). Tropical Atlantic variability: Patterns, mechanisms, and impacts. Geophys. Monogr. Ser..

[52] Becker E, Kirtman BP, Pegion K (2020). Evolution of the North American multi-model ensemble. Geophys. Res. Lett..

[53] Delgado JM (2018). Seasonal drought prediction for semiarid northeastern Brazil: Verification of six hydro-meteorological forecast products. Hydrol. Earth Syst. Sci..

[54] Hansen LK, Salamon P (1990). Neural network ensembles. IEEE Trans. Pattern Anal. Mach. Intell..

[55] Alobaidi MH, Ouarda TBMJ, Marpu PR, Chebana F (2021). Diversity-driven ANN-based ensemble framework for seasonal low-flow analysis at ungauged sites. Adv. Water Resour..

[56] Eissa Y, Gustafson B, Ghedira H, Bender G, Ouarda TBMJ (2014). Intercomparison of solar maps derived from an ensemble-ANN model and a semi-empirical model for a desert environment. Energy Procedia.

[57] Rumelhart D, Hinton G, Williams R (1986). Learning representations by back-propagating errors. Nature.

[58] Breiman L (1996). Bagging predictors. Mach. Learn..

[59] Wilks, D. S. *Statistical Methods in the Atmospheric Sciences*. (2019).

[60] Greene WH (2003). Econometric Analysis.

[61] Wilks DS (2011). Statistical Methods in the Atmospheric Sciences.

[62] Becker E, van den Dool H (2016). Probabilistic seasonal forecasts in the North American Multimodel Ensemble: A baseline skill assessment. J. Clim..

[63] Jolliffe IT, Stephenson DB (2012). Forecast Verification.

[64] Murphy AH (1973). A new vector partition of the probability score. J. Appl. Meteorol. Climatol..

[65] Efron B, Tibshirani R (1994). An Introduction to the Bootstrap.

[66] Uvo CB, Repelli CA, Zebiak SE, Kushnir Y (1998). The relationships between tropical Pacific and Atlantic SST and northeast Brazil monthly precipitation. J. Clim..

[67] Hounsou-Gbo GA (2019). SST indexes in the tropical South Atlantic for forecasting rainy seasons in Northeast Brazil. Atmosphere (Basel).

[68] Alves JMB, Kayano MT (1991). Estudo preliminar da precipitacao no Sul do Ceara durante a pre-estacao chuvosa. Climanálise—Boletim de Monitoramento e Análise Climática.

[69] Kharin VV, Zwiers FW (2003). Improved seasonal probability forecasts. J. Clim..

[70] van den Dool HM, Toth Z (1991). Why do forecasts for “near normal” often fail?. Weather Forecast.

[71] Kharin VV (2009). Skill assessment of seasonal hindcasts from the Canadian historical forecast project. Atmos. Ocean.

[72] Xing W, Wang B, Yim SY (2016). Long-lead seasonal prediction of China summer rainfall using an EOF-PLS regression-based methodology. J. Clim..

