# Supplementary material for: Short-lead seasonal precipitation forecast in northeastern Brazil using an ensemble of artificial neural networks
Source: Sci Rep. 2023 Nov 22;13:20429. doi: 10.1038/s41598-023-47841-y (PMC10665445; doi:10.1038/s41598-023-47841-y)
Supplement: Supplementary file 1 — Supplementary Information. [file 41598_2023_47841_MOESM1_ESM.pdf]

Short-Lead Seasonal Precipitation Forecast in Northeastern Brazil Using an Ensemble of Artificial Neural Networks

Enzo Pinheiro*[1], Taha B.M.J. Ouarda[1]

[1]*Institut National de la Recherche Scientifique, Centre Eau-Terre-Environnement, 490 de la Couronne, Québec (QC), G1K9A9, Canada;*

Correspondance: Institut National de la Recherche Scientifique, Centre Eau-Terre-Environnement, 490 de la Couronne, Office 2435, Québec (QC), G1K9A9, Canada.

Email: pinheiroenzo92@gmail.com

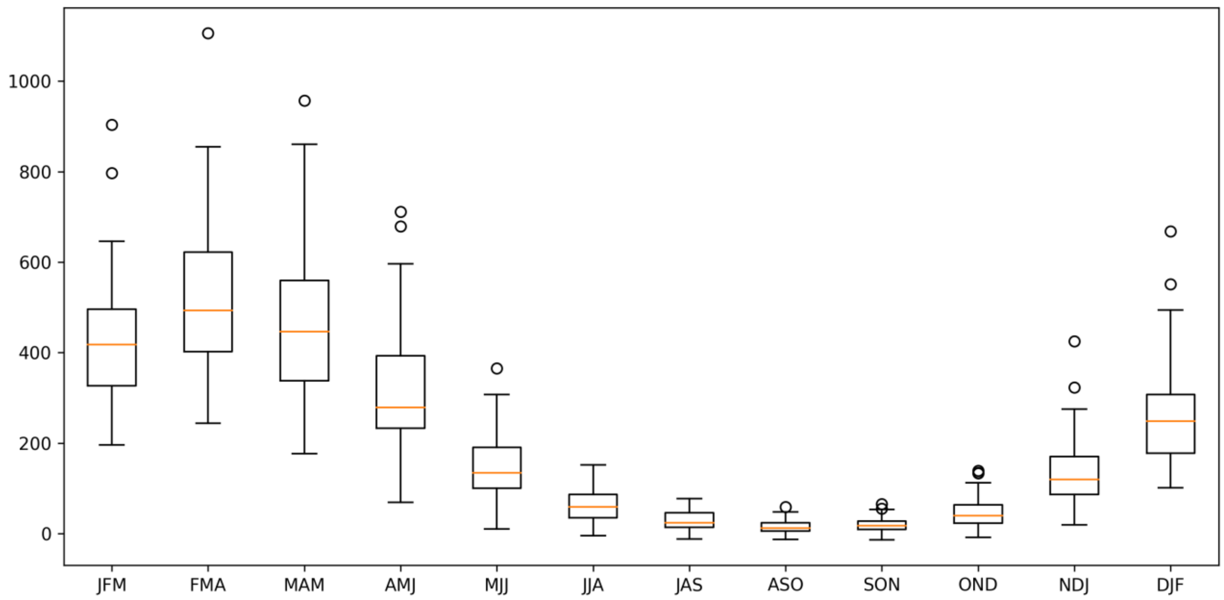

Supplementary Figure 1 – Interannual spatially-averaged precipitation variability (mm) from 1982-2021 over the state of Ceará for each season.

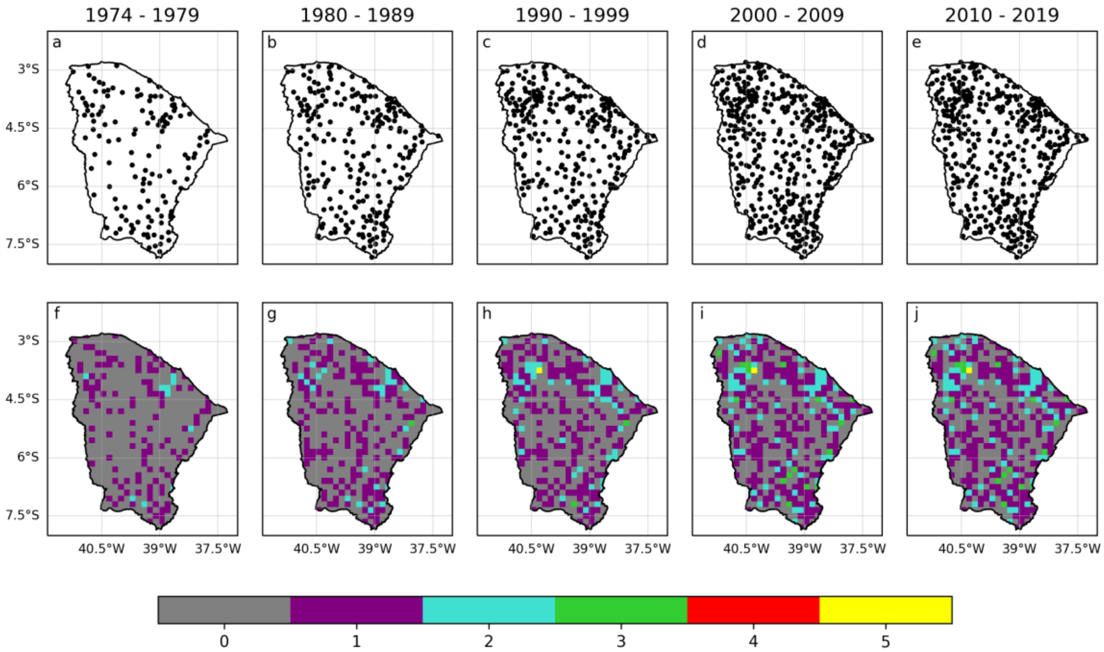

Supplementary Figure 2 – Geographical location of rain gauges (top) and number of stations per grid cell (bottom) in 10-year intervals.

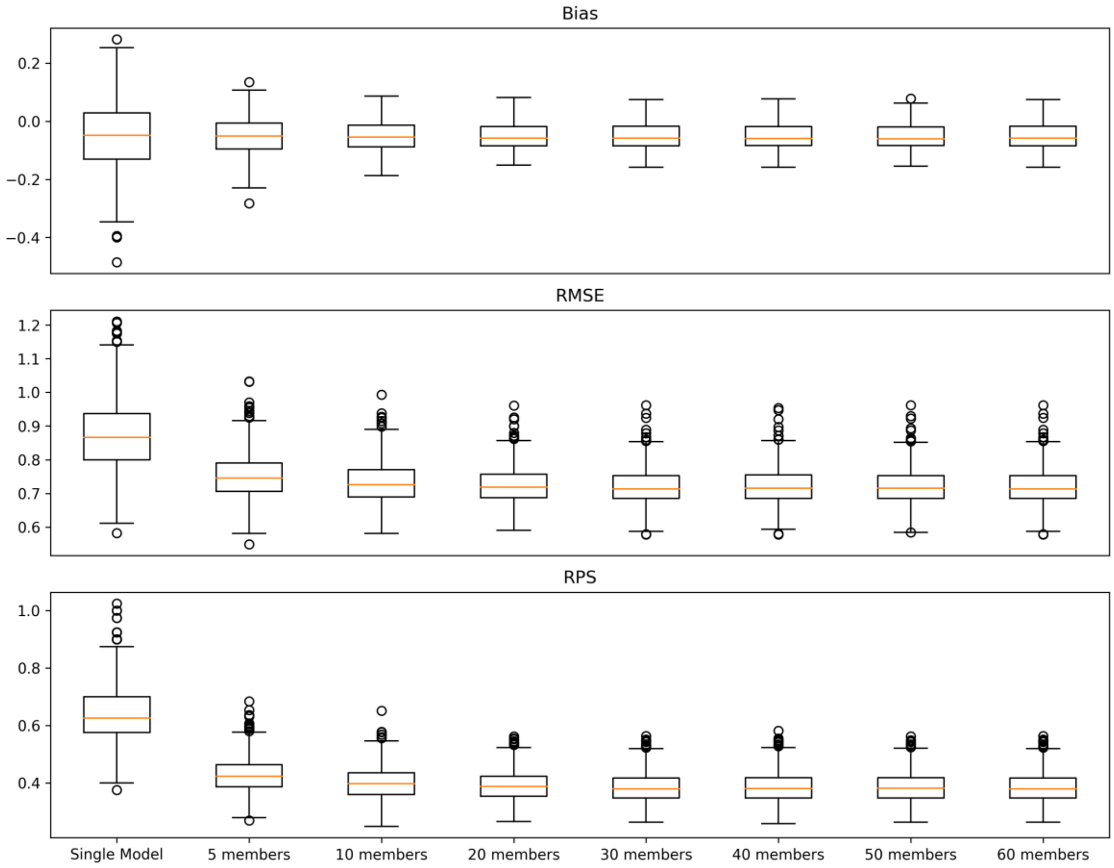

Supplementary Figure 3 – Cross-validation Bias (top panel, standardized units), RMSE (middle panel, standardized units) and RPS (bottom panel, unitless) distributions of the EANN with respect to ensemble sizes.

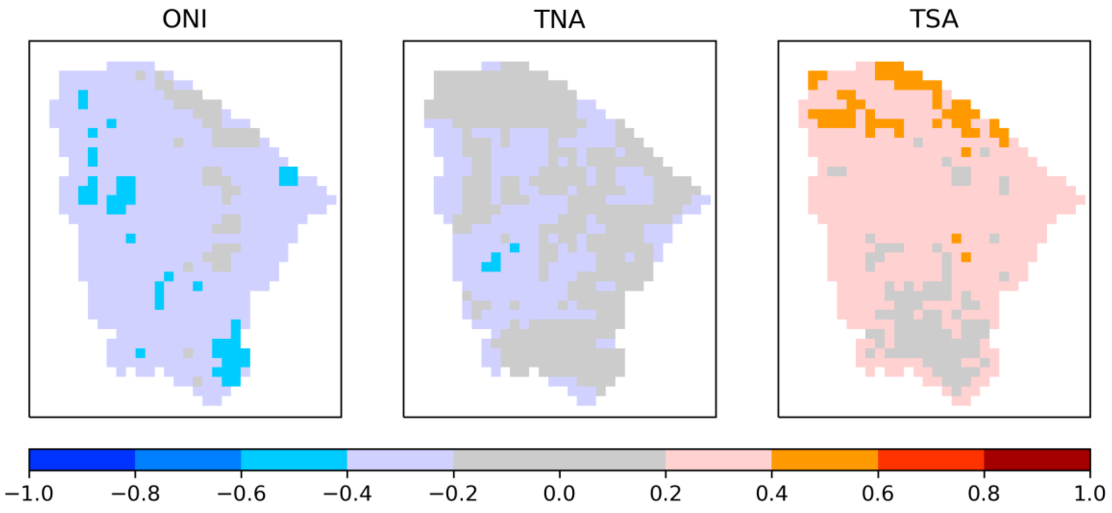

Supplementary Figure 4 – Spearman correlation between OND climate indices (ONI (left), TNA (center) and TSA (right)) and gridded FMA precipitation.

Model	Institution	Ensemble size
**CanCM4i**	Environmental Canada	10
**GEM-NEMO**	Centre de recherche en prévision numérique (RPN)	10
**CCSM4**	National Center for Atmospheric Research (NCAR)	10
**ECHAM4.6**	Max Planck Institute for Meteorology	20

Supplementary Table 1 – Description of the dynamical models.

	**EANN**	**ECHAM4.6**	**CanCM4i**	**MLR**	**GEM-NEMO**	**CCSM4**
**EANN**	1.00	**0.00**	0.06	0.07	**0.01**	**0.04**
**ECHAM4.6**	**0.00**	1.00	**0.00**	**0.00**	**0.00**	**0.00**
**CanCM4i**	0.06	**0.00**	1.00	0.89	**0.00**	0.89
**MLR**	0.07	**0.00**	0.89	1.00	**0.00**	0.77
**GEM-NEMO**	**0.01**	**0.00**	**0.00**	**0.00**	1.00	**0.00**
**CCSM4**	**0.04**	**0.00**	0.89	0.77	**0.00**	1.00

Supplementary Table 2 – The p-value of the two-sample *t*-test between models deterministic forecasts. The statistically significant values are shown in bold.

	NMME	NMME-EANN	NMME-EANN-ECHAM4.6
**NMME**	1.00	0.09	0.13
**NMME-EANN**	0.09	1.00	**0.00**
**NMME-EANN-ECHAM4.6**	0.13	**0.00**	1.00

Supplementary Table 3 – Same as Supplementary Table 1 but for MMEs.
